# Microglial NLRP3 inflammasome activation mediates IL-1β release and contributes to central sensitization in a recurrent nitroglycerin-induced migraine model

**DOI:** 10.1186/s12974-019-1459-7

**Published:** 2019-04-10

**Authors:** Wei He, Ting Long, Qi Pan, Shanshan Zhang, Yixin Zhang, Dunke Zhang, Guangcheng Qin, Lixue Chen, Jiying Zhou

**Affiliations:** 1 Department of Neurology, The First Affiliated Hospital of Chongqing Medical University, 1st You Yi Road, Yu Zhong District, Chongqing, 400016 People’s Republic of China; 2 Laboratory Research Center, The First Affiliated Hospital of Chongqing Medical University, 1st You Yi Road, Yu Zhong District, Chongqing, 400016 People’s Republic of China

**Keywords:** Chronic migraine, Nod-like receptor protein 3 inflammasome, Interleukin-1β, Central sensitization

## Abstract

**Background:**

Central sensitization is an important mechanism of chronic migraine (CM) and is related to the inflammatory response of microglia. The NOD-like receptor protein 3 (NLRP3) inflammasome may regulate the inflammatory process of microglia in several neurological diseases, but its role in CM is largely unknown. Therefore, the aim of this study was to identify the precise role of microglial NLRP3 in CM.

**Methods:**

An experimental CM mouse model was established by repeated intraperitoneal (i.p) injection with nitroglycerin (NTG). We evaluated the expression levels of NLRP3 and its downstream interleukin (IL)-1β protein in the trigeminal nucleus caudalis (TNC; which is a central area relevant to migraine pain) at different time points. To further examine the effects of the NLRP3 inflammasome pathway on central sensitization of CM, we examined MCC950, an NLRP3 inflammasome-specific inhibitor, and IL-1ra, an IL-1β antagonist, whether altered NTG-induced mechanical hyperalgesia of the periorbital area and hind paw. The effect of MCC950 and IL-1ra on c-Fos, phosphorylated extracellular signal-regulated kinase (p-ERK) and calcitonin gene-related peptide (CGRP) expression in the TNC were also analyzed. The cell localization of NLRP3 and IL-1β in the TNC was evaluated by immunofluorescence staining.

**Results:**

Repeated NTG administration induced acute and chronic mechanical hyperalgesia and increased expression of NLRP3 and IL-1β. Blockade of NLRP3 or IL-1β reduced NTG-induced hyperalgesia, and this effect was accompanied by a significant inhibition of the NTG-induced increase in p-ERK, c-Fos and CGRP levels in the TNC. Immunofluorescence staining revealed that NLRP3 and IL-1β were mainly expressed in microglia in the TNC, and the IL-1β receptor, IL-1R, was mainly expressed in neurons in the TNC.

**Conclusions:**

These results indicate that NLRP3 activation in the TNC participates in the microglial-neuronal signal by mediating the inflammatory response. This process contributes to the central sensitization observed in CM.

**Electronic supplementary material:**

The online version of this article (10.1186/s12974-019-1459-7) contains supplementary material, which is available to authorized users.

## Introduction

Chronic migraine (CM) is a complex neurological disease which usually progresses from episodic migraine [1], and the global prevalence is 0.5–1% [2]. Due to limited treatment options and effects, more than half of patients with CM reported dissatisfaction with their treatments [3]. A better understanding of the molecular mechanism of CM will provide new therapeutic approaches.

The current theory suggests that CM may be due to the chronic sensitization of central pain pathways caused by repeated migraine attacks [4]. Among these pathways, the activation of the trigeminal nucleus caudalis (TNC) area is an important manifestation of central sensitization [4]. In addition to neurons, microglia and the inflammatory factors they secrete are also involved in the regulation of central sensitization [5, 6]. This involvement has been extensively demonstrated in other pain models [7]. In recent years, the role of microglia in migraine has also been preliminarily understood [8]. According to our previous study, microglia were associated with central sensitization in CM [9]. However, the specific intracellular mechanism is unclear, which is the current focus of our research.

Based on accumulating evidence, IL-1β is involved in migraine pathology. For example, during a migraine attack and the interictal periods, IL-1β markedly increases in peripheral blood [10]. In the trigeminal ganglion (TG), IL-1β mediates the pro-inflammatory process, contributes to the activation of the trigeminal satellite cells and promotes cross excitation of satellite glial cells and neurons [11]. However, these studies focused on the peripheral area. In the central area of the trigeminovascular pathway, especially the TNC, which is the important area of the development and maintenance of CM, the mechanism of action of IL-1β is largely unknown.

Considerable evidence from animal models of neuroinflammation suggests that IL-1β of the central nervous system (CNS) is mainly from microglia [12]. The maturation of IL-1β has a unique molecular mechanism in a kind of intracellular multiprotein complex, the NOD-like receptor protein3 (NLRP3) inflammasome. The NLRP3 inflammasome is an innate immune complex that mediates the activation of caspase-1, which in turn cleaves pro-IL-1β to form mature IL-1β [13]. This function is crucial for the regulation of neuroinflammation mediated by microglia.

The NLRP3 inflammasome has been associated with several inflammatory disorders of the CNS, such as depression [14], spontaneous intracerebral haemorrhage [15] and multiple sclerosis [16]. However, the role of NLRP3 in CM has not been reported. Notably, the conditions for NLRP3 activation, such as the high concentrations of extracellular K+ ions [17, 18], increased peroxynitrite, mitochondrial dysfunction and the generation of reactive oxygen species (ROS), usually underlie the generation of a migraine [19–23].

Therefore, investigating the role of the NLRP3 inflammasome in CM-associated pathology and functional outcomes is intriguing. We hypothesized that the NLRP3 inflammasome has the potential to induce IL-1β-related neuroinflammation in CM. Continuous inflammatory processes contribute to central sensitization.

In this study, we aimed to investigate the role of the NLRP3/IL-1β pathway in nitroglycerin (NTG)-induced hyperalgesia and its underlying mechanism in the TNC. We expect to identify an effective therapeutic approach for CM.

## Materials and methods

### Animals

The subjects were male C57BL6/J mice, weighing 18–25 g and aged 8–12 weeks. The mice were provided by the Experimental Animal Center of Chongqing Medical University (Chongqing, China). Mice were maintained at a temperature of 22 ± 2 °C with a humidity of 50 ± 5% on a 12-h light–dark cycle, and food and water were freely available. All procedures involving the use of animals were approved by the Animal Care and Use Committee at Chongqing Medical University in China. Animal experiments were also carried out in accordance with the ethical guidelines of the International Association for the Study of Pain for investigations of experimental pain in conscious animals [24]. All experiments were performed in a blinded manner, and mice were habituated for 1 week before any experimental procedures were initiated. Animals were weighed daily during treatment, and no adverse effects of treatment were observed.

### Establishment of a chronic migraine model

The procedures listed below were performed as described in the report by Pradhan et al. [25]. We established a CM model by repeated intraperitoneal (i.p) injection with NTG. NTG was purchased from the First Affiliated Hospital of Chongqing Medical University (Chinese Drug Approval Number: H11020289; 5.0 mg/ml). No significant differences in the mechanical thresholds were observed between animals injected with 0.9% saline and the NTG solvent (6% propylene glycol, 6% alcohol, and 0.9% saline) [25]; therefore, the vehicle control used in the experiments was 0.9% saline. NTG was freshly diluted in 0.9% saline to a dose of 10 mg/kg. All injections were administered at a volume of 10 ml/kg. The model group received an NTG injection every other day for a total of five times over 9 days (in the chronic treatment experiment, the injections were performed five times, on days 3, 5, 7, 9 and 11).

### Intracerebroventricular cannulation

Intracerebroventricular (I.C.V.) cannulation was performed as described previously [26]. Mice were anaesthetized with 10% chloral hydrate (4 ml/kg, i.p.) and positioned in a stereotaxic apparatus (ST-51603; 30 Stoelting Co., Chicago, IL, USA). A 26-gauge stainless steel cannula was placed in the left lateral cerebral ventricle at predetermined coordinates (lateral 1.6 mm and anteroposterior 1 mm to bregma and horizontal 2 mm from the dura mater) [27]. The cannula was secured using dental acrylic. Mice were provided a minimum of 5 days to recover before any treatment or behavioural test.

### Treatment drug administration

Experiment 2 used the NLRP3 inhibitor MCC950 (Thermo, Houston, TX, USA), which has a specific and strong inhibitory effect on NLRP3 [28, 29]. After i.p. injection, MCC950 can pass through the blood-brain barrier to act on the central area [29]. MCC950 was dissolved in sterile phosphate-buffered saline (PBS) at a concentration of 10 mg/ml, and each mouse was given a dose of 10 mg/kg with i.p. injection once a day for 11 days. The doses for MCC950 used in the experiment were based on previous studies [29]. Sterile PBS of an equal volume was used as the control.

Experiment 3 used an IL-1 receptor antagonist, IL-1ra (PeproTech, Rocky Hill, NJ, USA). IL-1ra was dissolved in sterile PBS at a concentration of 2 μg/μl, and each mouse was given a 4 μg I.C.V. injection once a day for 11 days. The doses used in this experiment were based on previous studies [27]. Sterile PBS of an equal volume was used as the control.

### Sensory sensitivity testing

We used the von Frey test to examine the mechanical sensitivity, the threshold for responses to punctuate mechanical stimuli (mechanical hyperalgesia). The tests used the electronic von Frey device (Electrovonfrey, IITC Inc., Woodland Hills, CA, USA) that automatically recorded thresholds after stimulation. The investigators were blinded to the experimental groups. All the mice were habituated to the test chamber before testing began.

Before testing the periorbital sensitivity, the mice were placed into a 9-cm-long restraining glass cylinder such that only the head poked out. The restrainer allowed head and forepaw movements but prevented the animals from turning in the cylinder. Then, following application of an electronic von Frey monofilament to the periorbital area of the face over the rostral portion of the eye, a positive response was defined as when the mouse stroked its face with the ipsilateral forepaw, quickly retracted its head from the stimulus, or vocalized. To test the hind paw sensitivity, we placed mice in wire mesh boxes, applying an electronic von Frey monofilament to the plantar surface of the animal hind paw from the underside of the mesh stand. A positive response with hind paw was defined as withdrawal, shaking, or licking of the paw in response to stimulation. Each site was tested at least three times with an interval of at least 1 min.

In experiment 1, days 1, 3, 5, 7 and 9 were testing days. Mice were tested for mechanical thresholds before injection (baseline responses) and 2 h after injection with NTG or vehicle.

In experiments 2 and 3, days 3, 5, 7, 9 and 11 were testing days. The treatment of the mice on testing days was as follows: all mice were first tested for baseline mechanical thresholds, followed by injection of preventative therapy (MCC950 or IL-1ra) or their respective vehicles; then administration of NTG after 30–45 min; and finally, at 2-h post-NTG injection, mice were tested again for mechanical responses.

An additional figure file shows this procedure in more detail [see Additional file 1].

### Quantitative real-time reverse trancriptase polymerase chain reaction (qRT-PCR)

Mice were anesthetized and decapitated 24 h after the last NTG or saline injection, and the TNC tissues were obtained and immediately stored in liquid nitrogen until analysis. Total RNA was purified using an RNAiso Plus reagent (Takara, Tokyo, Japan), and yield and purity were assessed spectrophotometrically with a NanoDrop spectrophotometer (Thermo, Waltham, MA, USA). cDNAs were synthesized using a PrimeScript™ RT Reagent Kit (Takara). Gene-specific primers were obtained from TSINGKE Biological Technology (Chengdu, China), and specificity was tested by basic local alignment search tool (BLAST). qRT-PCR was performed with SYBR® Premix Ex Taq™ II (Takara) using a CFX96 Touch thermocycler (Bio-Rad, Hercules, CA, USA). All final expression data were expressed as a target/reference ratio in experimental samples and normalized to the target/reference ratio of the calibrator. At the end of each run, melting curve analysis was performed to verify the single-product detection of the primers. All fluorescence data were processed by a post-PCR data analysis software program, and relative gene expression was normalized to the internal reference glyceraldehyde 3-phosphate dehydrogenase (GAPDH) using the 2^−∆∆CT^ method [30]. Sequence-specific primers for NLRP3, IL-1β and GADPH are shown in Table 1.

**Table 1:** Sequences of primers used

Gene	Forward primer	Reverse primer
NLRP3	CCATCAATGCTGCTTCGACA	GAGCTCAGAACCAATGCGAG
IL-1β	GTGAAATGCCACCTTTTGACAGTGA	GAAGGTCCACGGGAAAGACAC
GAPDH	AGACAGCCGCATCTTCTTGT	TGATGGCAACAATGTCCACT

### Western blot analysis

For experiment 1 to detect the NLRP3 and IL-1β proteins, TNCs were collected 24 h after different NTG injections. For experiments 2 and 3, TNCs were collected 24 h after the last NTG/saline injection to detect the levels of NLRP3, IL-1β and calcitonin gene-related peptide (CGRP) proteins, and TNCs were collected 2 h after the last NTG/saline injection to detect levels of the phosphorylated extracellular signal-regulated kinase (p-ERK) and c-Fos proteins. Mice were anesthetized and decapitated, and fresh samples were homogenized in buffer containing protease and phosphatase inhibitor cocktails (Beyotime, Shanghai, China). Protein concentrations were determined using a bicinchoninic acid (BCA) Protein Assay kit (Beyotime). Equal amounts of protein samples (40 μg) were loaded on sodium dodecyl sulfate polyacrylamide gel electrophoresis (SDS PAGE) gels, electrophoresed and transferred to a nitrocellulose membrane. Membranes were blocked with 5% non-fat milk in Tris-buffered saline (TBS) containing 0.1% Tween 20 for 2 h at room temperature and incubated with each antibody at 4 °C overnight. The primary antibodies were anti-NLRP3, anti-IL-1β, anti-c-Fos, anti-p-ERK and anti-CGRP antibodies [see Table 2]. An anti-β-actin antibody was used as the loading control. Then, horseradish peroxidase conjugated secondary antibodies were applied for 2 h at room temperature. The membranes were probed with a BeyoECL Plus kit (Beyotime). The ImageJ analysis system (Fusion, Germany) was used to quantify the specific bands.

**Table 2:** Antibodies used in Western blot analysis, immunofluorescence staining and immunohistochemistry

Antibody	Manufacturer	Catalogue number	Host	Dilution
For Western blot analysis				
NLRP3	Abcam, UK	ab205680	Rat	1:1000
IL-1β	Santa Cruz, USA	sc-515,598	Mouse	1:3000
c-Fos	Santa Cruz, USA	sc-447	Mouse	1:1200
p-ERK	Cell Signaling, USA	5726	Mouse	1:1000
CGRP	Abcam, UK	ab10062	Mouse	1:2000
β-actin	Proteintech, China	20,536–1-AP	Rabbit	1:9000
Anti-rabbit IgG (HRP)	ZSGB-BIO, China	ZB-2301	Goat	1:5000
Anti-mouse IgG (HRP)	ZSGB-BIO, China	ZB-2305	Goat	1:5000
Anti-rat IgG (HRP)	ZSGB-BIO, China	ZB-2307	Goat	1:5000
For immunofluorescence staining				
NLRP3	Biorbyt	orb101128	Rabbit	1:400
CGRP	Santa Cruz, USA	sc-57,053	Mouse	1:100
IL-1β	Santa Cruz, USA	sc-515,598	Mouse	1:50
IL-1R	Abcam, UK	ab124962	Rabbit	1:200
Iba-1	Santa Cruz, USA	sc-32,725	Mouse	1:200
Iba-1	Wako, Japan	019–19,741	Rabbit	1:500
GFAP	Abcam, UK	ab10062	Mouse	1:100
NeuN	Abcam, UK	ab104224	Mouse	1:500
Alexa Fluor 488 goat anti-rabbit IgG	Beyotime, China	A0423	Goat	1:400
Alexa Fluor 488 goat anti-mouse IgG	Beyotime, China	A0428	Goat	1:400
Cy3 goat anti-rabbit IgG	Beyotime, China	A0516	Goat	1:400
Cy3 goat anti-mouse IgG	Beyotime, China	A0521	Goat	1:400
For immunohistochemistry				
c-Fos	Abcam, USA	ab208942	Mouse	1:1000

### Immunofluorescence (IF) staining

For ionized calcium binding adaptor molecule 1 (Iba-1), NLRP3, IL-1β and CGRP detection, tissues were collected 24 h after the last NTG or vehicle injection. For p-ERK detection, tissues were collected at 2 h after the last NTG or vehicle injection. Mice were anesthetized and perfused intracardially with 15 ml ice-cold 0.1 M PBS (pH 7.2) and then with 50 ml ice-cold 4% paraformaldehyde (PFA)/0.1 M PBS. Whole brains, including TNCs, were post-fixed overnight with 4% PFA/0.1 M PBS at 4 °C. The tissues were cryoprotected in 30% sucrose/0.1 M PBS for 24–36 h or until the tissue sank. Brain tissues were flash frozen in 2-methyl butane on dry ice, and coronal sections of the TNC were sliced at 10 mm. All sections within bregma at − 7.47 mm to − 8.24 mm were collected [31].

Immunofluorescence and double immunostaining were performed on cryofixed sections. The sections were incubated at 4 °C overnight with primary antibodies, as listed in Table 2, antibodies against NLRP3, CGRP, IL-1β, and IL-1R and antibodies against Iba-1, glial fibrillary acidic protein (GFAP) and neuronal nuclei (NeuN) (used to mark microglia, astrocytes and neurons, respectively). We used 4′,6-diamidino-2-phenylindole (DAPI) for nuclear staining. Alexa Fluor 488 and Cy3-labelled goat anti-mouse or goat anti-rabbit immunoglobulin (IgG) were used as secondary antibodies (see Table 2). Slides containing the negative controls were incubated with TBS instead of primary antibodies, which resulted in no positive labeling of the tissues.

#### Immunofluorescence imaging data analysis

The TNC area was determined based on morphological appearance under a light microscope using the Mouse Brain Atlas as a reference [31]. Sections were viewed under a confocal laser scanning fluorescence microscope (ZEISS, Oberkochen, Germany). Laminae I-V of the TNC was determined manually as the area of interest using a rectangular tool. A significant difference in the area of the TNC was not observed among the experimental groups. Images were analyzed by using Image-Pro Plus 6.2 software (Bethesda, MD, USA). The ratio of the cross-sectional area immunoreactive for Iba-1 and p-ERK were calculated in the TNC at × 20 magnification. The fluorescence intensity of CGRP was presented by mean optical density (OD) in the TNC at × 10 magnification. TNCs in both hemispheres from six sections per animal (*n* = 6 mice/group) were included in the analysis. All evaluations were performed by an observer who was blinded to the experimental groups.

### Immunohistochemistry (IH) staining

For c-Fos detection, sections were incubated with 0.3% H_2_O_2_ for 30 min and blocked with 10% normal goat serum for 2 h. These sections were incubated overnight at 4 °C with a primary antibody against c-Fos (Table 2) and then incubated with a secondary antibody solution (biotinylated goat anti-mouse antibody; ZSGB-BIO, Beijing, China) for 2 h at room temperature. Next, sections were incubated in horseradish enzyme-labelled streptomyces lecithin reagent (ZSGB-BIO) for 1 h at room temperature. Nickel-enhanced diaminobenzidine (DAB) was used to visualize primary antibody staining. Sections were mounted, dried and dehydrated.

#### Immunohistochemistry imaging data analysis

The TNC area of interest in the images of each section was determined as previously described. Images were observed under a ZEISS Axio Imager A2 microscope using a × 20 objective lens. Images were analyzed using Image-Pro Plus 6.0 software. The expression of c-Fos was blindly examined and quantified by counting c-Fos-positive cells from averaging six TNC sections per mouse (*n* = 6 mice/group).

### Statistical analyses

All data are presented as the means ± standard deviations (SDs). All statistical analyses and graphs were performed using GraphPad Prism version 6.0 (GraphPad Software Inc., San Diego, CA, USA). Statistical analysis of the mechanical threshold data was performed using two-way repeated measures analysis of variance (ANOVA), with drug and time as factors. Western blot data were analyzed by one-way ANOVA followed by Tukey’s multiple comparison test. For IF, immunohistochemical and qRT-PCR experiments, data were analyzed using unpaired *t* test (if the variances were significantly different, unpaired *t* test with Welch’s correction was used.) In all statistical comparisons, a *P* value< 0.05 was considered significant.

#### Experimental design

An additional figure file shows this process in more detail [see Additional file 1].

### Experiment 1

This experiment was performed to confirm whether repeated NTG stimulation could cause migraine-associated hyperalgesia in mice and form a central sensitization state. This experiment was also performed to verify whether NLRP3 and its related pathways were involved in this process.

Animals were given NTG or saline injections every other day (on days 1, 3, 5, 7 and 9) and tested of basal mechanical responses and NTG post-treatment responses (details in the “Materials and methods” section). The expression levels of the NLRP3 and IL-1β proteins were detected by Western blotting 24 h after the each NTG or saline injections. Two hours after the last NTG or saline injection, we tested c-Fos protein levels by IH and tested p-ERK levels by IF. At 24 h after the last NTG or saline injection, we tested NLRP3 and IL-1β mRNA levels by qRT-PCR and tested Iba-1 (microglial maker) and CGRP protein levels in the TNC by IF, as well as double IF for Iba-1, NLRP3 and IL-1β.

### Experiment 2

This experiment was performed to test the preventative effect of NLRP3 inhibition on NTG-induced migraine-associated pain.

A specific small molecule inhibitor of the NLRP3 inflammasome, MCC950, was used. The following groups were set up for the experiment: saline group, NTG group, NTG+MCC950 group and NTG+vehicle (VEH) group. For the saline or NTG group, mice were given saline or NTG injections on days 3, 5, 7, 9 and 11. For the NTG+MCC950 or NTG+VEH group, mice were injected once daily with MCC950 (10 mg/kg, i.p.) or VEH (equal volumes of PBS) for 11 days. On days 3, 5, 7, 9 and 11, mice were injected with NTG after being injected MCC950 or VEH. All mice were tested for basal mechanical responses and NTG post-treatment responses (details in the “Materials and methods” section). The levels of the IL-1β and CGRP proteins were detected by Western blotting 24 h after the NTG injection on day 11. The levels of the c-Fos and p-ERK proteins were detected by Western blotting 2 h after NTG injection on day 11.

### Experiment 3

This experiment was performed to test the preventative effect of IL-1β blockage on NTG-induced migraine-associated pain.

An IL-1 receptor antagonist, IL-1ra, was used to block IL-1β. The following groups were established for the experiment: saline group, NTG group, NTG+IL-1ra group and NTG+VEH group. For the saline or NTG group, mice were given saline or NTG injections on days 3, 5, 7, 9 and 11. For the NTG+IL-1ra or NTG+VEH group, mice underwent I.C.V. cannulation and were I.C.V. injected with IL-1ra (4 μg/mouse/day) or VEH (equal volumes of sterile PBS) once daily for 11 days. On days 3, 5, 7, 9 and 11, mice were injected with NTG after being injected with IL-1ra or VEH. All mice were tested for basal mechanical responses and NTG post-treatment responses (details in the “Materials and methods” section). The levels of NLRP3 and CGRP proteins were detected by Western blotting 24 h after the NTG injection on day 11. The levels of c-Fos and p-ERK proteins were detected by Western blotting 2 h after NTG injection on day 11. The tissue for double IF analysis of the IL-1 receptor (IL-1R) with NeuN, Iba-1 and GFAP was collected 24 h after the NTG injection on day 11.

## Results

### Repeated NTG stimulation induced mechanical hyperalgesia

**Fig. 1:**
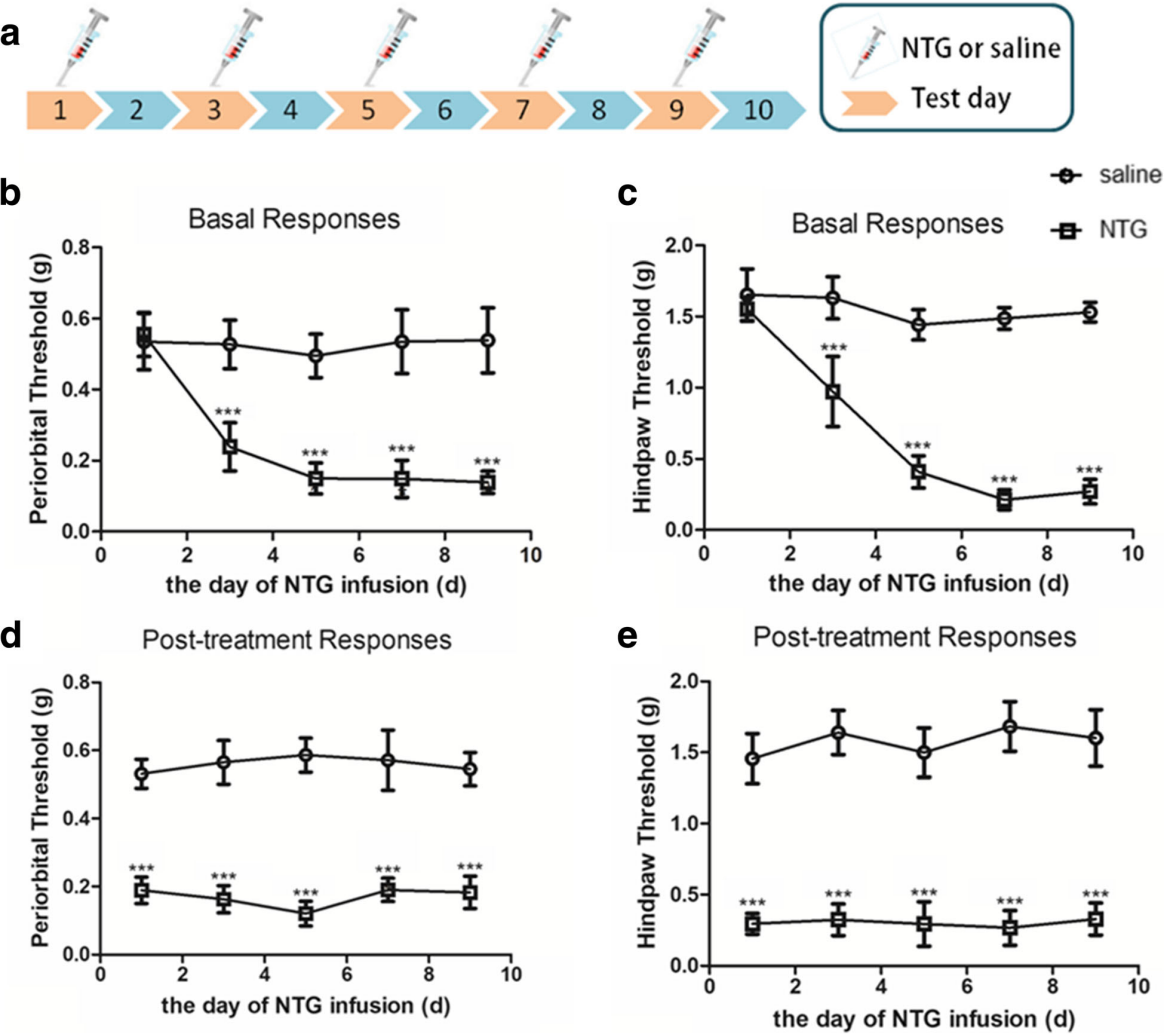
Repeated NTG administration induced basal hypersensitivity and acute hyperalgesia. **a** Drug administered in the first experiment. C57Bl/6J mice were administered nitroglycerin (NTG; 10 mg/kg, i.p.) every 2 days for 9 days, and saline as control. **b**, **c** Basal mechanical responses of periorbital area and hind paw, as assessed prior to NTG or saline administration, significantly decreased in the NTG group. Statistical analyses were performed by two-way ANOVA; ***p < 0.001 (*n* = 8 mice per group). **d**, **e** NTG administration produced significant hyperalgesia of periorbital area and hind paw in mice tested 2 h after NTG. Statistical analyses were performed by two-way ANOVA, ***p < 0.001 (*n* = 8 mice per group)

For experiment 1, mice were administered NTG or saline every other day (Fig. 1a). The baseline mechanical thresholds before administration were not significantly different between the two groups (*p* > 0.05; Fig. 1b, c date on the first day). However, the baseline mechanical thresholds of the hind paw and periorbital area were significantly decreased in the NTG group compared with those in the control (saline) group after the first injection (*p* < 0.001; Fig. 1b, c). Repeated administration of NTG maintained the reduction in the baseline mechanical threshold, which was most obvious on days 7 and 9. In addition, compared to the saline group, each NTG administration induced significant post-treatment (acute) hyperalgesia (*p* < 0.001; Fig. 1d, e).

### Repeated NTG stimulation increased expression of the c-Fos, p-ERK and CGRP in the TNC

**Fig. 2:**
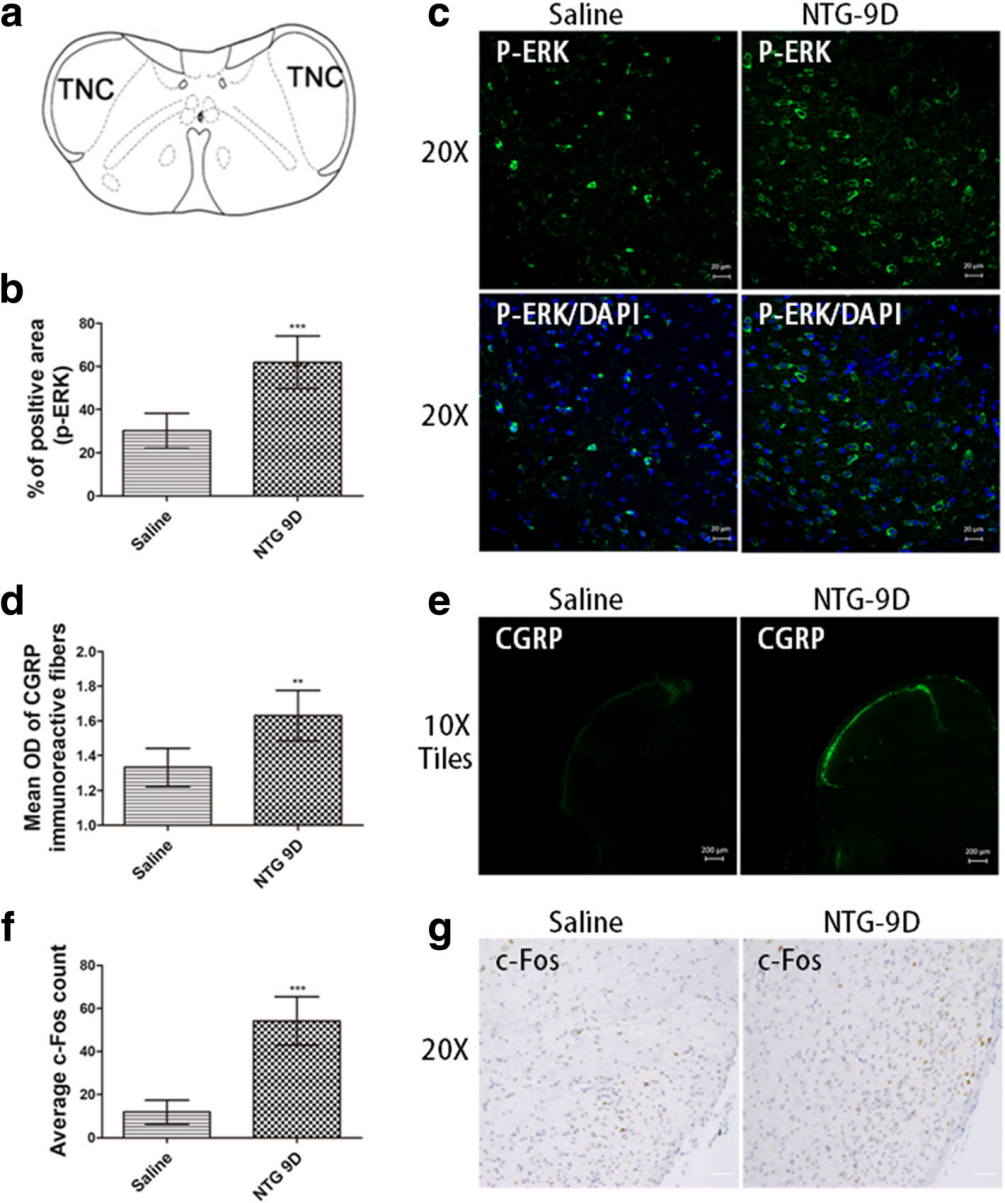
Repeated NTG administration increased the expression of migraine-related proteins in the TNC, such as c-Fos, p-ERK and CGRP. **a** Coronal view showing the location of the TNC in the mouse brain, which is the region of interest. **b** The ratio of the p-ERK immunoreactive area to the total area of the TNC was increased in the group treated with NTG (NTG-9D group). Unpaired *t* test, ***p < 0.001 versus the saline group (*n* = 6 mice per group). **c** Changes in p-ERK immunoreactivity in the TNC after repeated nitroglycerin (NTG) or saline administration for 9 days; × 20 objective lens; scale bar, 20 μm. **d** Repeated NTG administration increased the mean OD of the CGRP-immunoreactive fibres. Unpaired *t* test, ***p < 0.001 compared with the saline group (*n* = 6 mice per group). **e** Changes in CGRP immunoreactivity in the TNC after repeated NTG or saline administration for 9 days; × 10 objective lens; scale bar, 200 μm. **f** Quantification of the average number of c-Fos particles showed that repeated NTG administration significantly increased c-Fos expression in the TNC. Unpaired *t* test, ***p < 0.001 compared with the saline group (*n* = 6 mice per group). **g** Change in c-Fos immunoreactivity in the TNC after repeated NTG or saline administration for 9 days; × 20 objective

The activation of c-Fos and the phosphorylation of ERK have been used as a molecular reliable marker for neurons activation and central sensitization [32]. Our study have shown that repeated NTG stimulation induced significant increases in the protein expression levels of the p-ERK and c-Fos in the TNC (Fig. 2b, c, f, g). Based on these results, NTG induced acute hyperalgesia, which may be associated with the activation of c-Fos and p-ERK in headache-related brain regions (the TNC regulates craniofacial pain). CGRP is considered an endogenous migraine generator and plays a critical role in the initiation and maintenance of a migraine [33]. Repeated NTG stimulation reliably induced a significant basal hypersensitivity. To determine whether the basal hypersensitivity induced by repeated NTG administration corresponded to increased CGRP expression, we determined CGRP expression 24 h after the final administration of NTG in the TNC and observed significantly increased (Fig. 2d, e).

### Repeated NTG stimulation induced NLRP3 inflammasome activation

Consistent with our previous published study [9], the number of Iba-1-marked microglia cells was increased in the TNC after repeated administration of NTG. An additional figure file shows this finding in more detail [see Additional file 2]. We speculated that microglial activation correlated with the NTG-induced hyperalgesia. Hence, we investigated the role of the relevant pathways of microglia, such as the NLRP3 inflammasome pathway in CM-associated pathology and function.

**Fig. 3:**
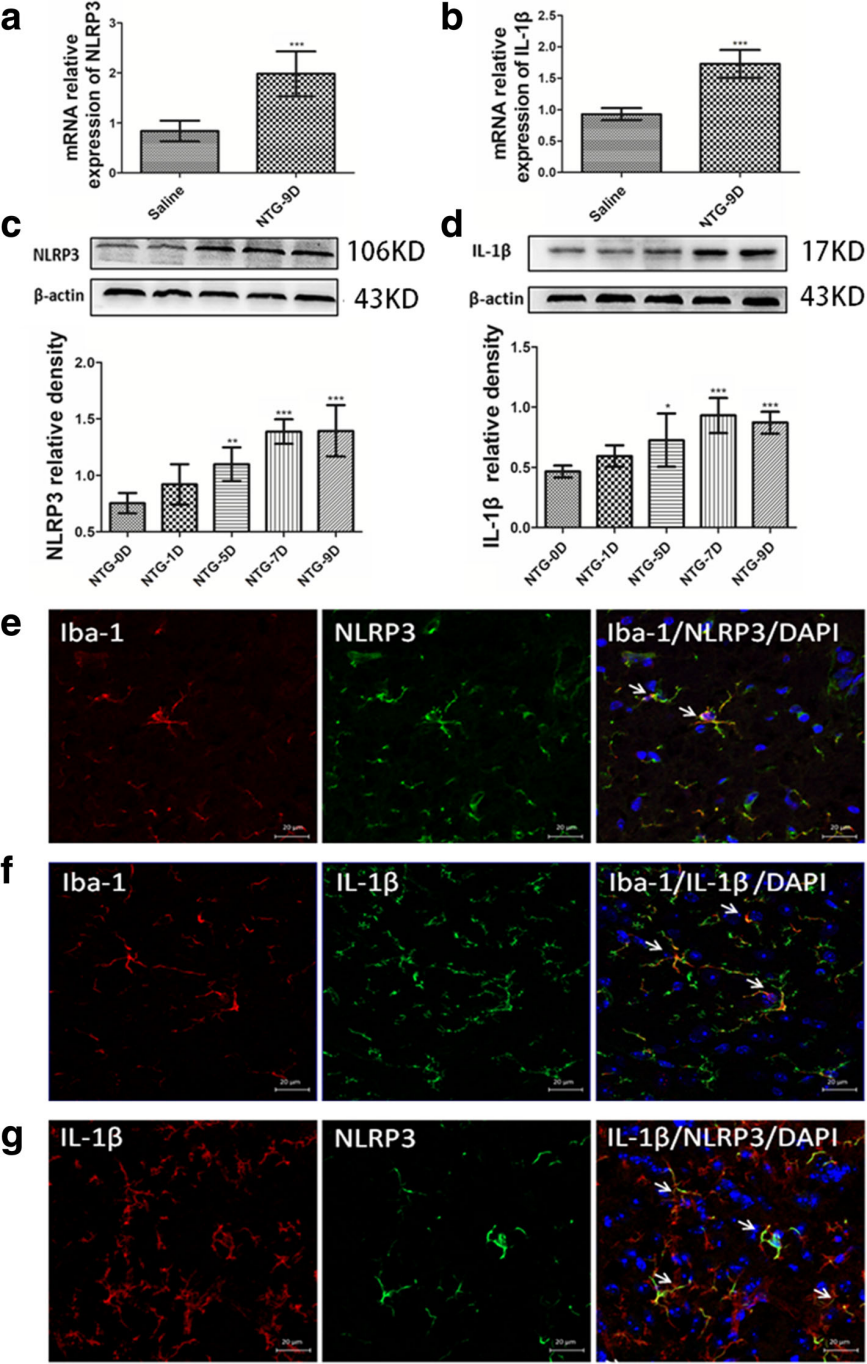
Repeated NTG administration increased the expression of NLRP3 and IL-1β on microglia. **a**, **b** After repeated nitroglycerin (NTG) or saline administration, NLRP3 and IL-1β mRNA expression analysis were tested by qRT-PCR. Unpaired *t* test, ***p < 0.001 compared with the saline group (*n* = 6 mice per group). **c**, **d** The NLRP3 and IL-1β protein expression were tested by western blot on different days (NTG-0D, namely before the first injection of NTG). One-way ANOVA and Tukey’s multiple comparison test, *p < 0.05, **p < 0.01, ***p < 0.001 versus the NTG-0D group (*n* = 6 per mice group). **e** Double immunofluorescence staining for Iba-1 (red) with NLRP3 (green) in the mouse TNC after repeated NTG administrations. The white arrow shows the colocalization of Iba-1 and NLRP3 proteins. **f** Double immunofluorescence staining for Iba-1 (red) and IL-1β (green). The white arrow shows the colocalization of IL-1β and Iba-1 proteins. **g** Double immunofluorescence staining of IL-1β (red) and NLRP3 (green). The white arrow shows the colocalization of the IL-1β and NLRP3 protein. **e**–**g** × 40 objective lens; scale bars, 20 μm

Our data shows that compared to repeated saline administration mice (control group), repeated NTG administration increased the mRNA and protein expression of NLRP3 and IL-1β in the TNC (*p* < 0.05; Fig. 3a–d). Moreover, double immunostaining indicated that NLRP3 and IL-1β were mainly expressed in microglia, and NLRP3 and IL-1β were co-localized in microglia in the TNC (Fig. 3e–g).

### NLRP3 inhibitor MCC950 attenuated NTG-induced hyperalgesia and expression of CGRP, c-Fos and p-ERK in the TNC

**Fig. 4:**
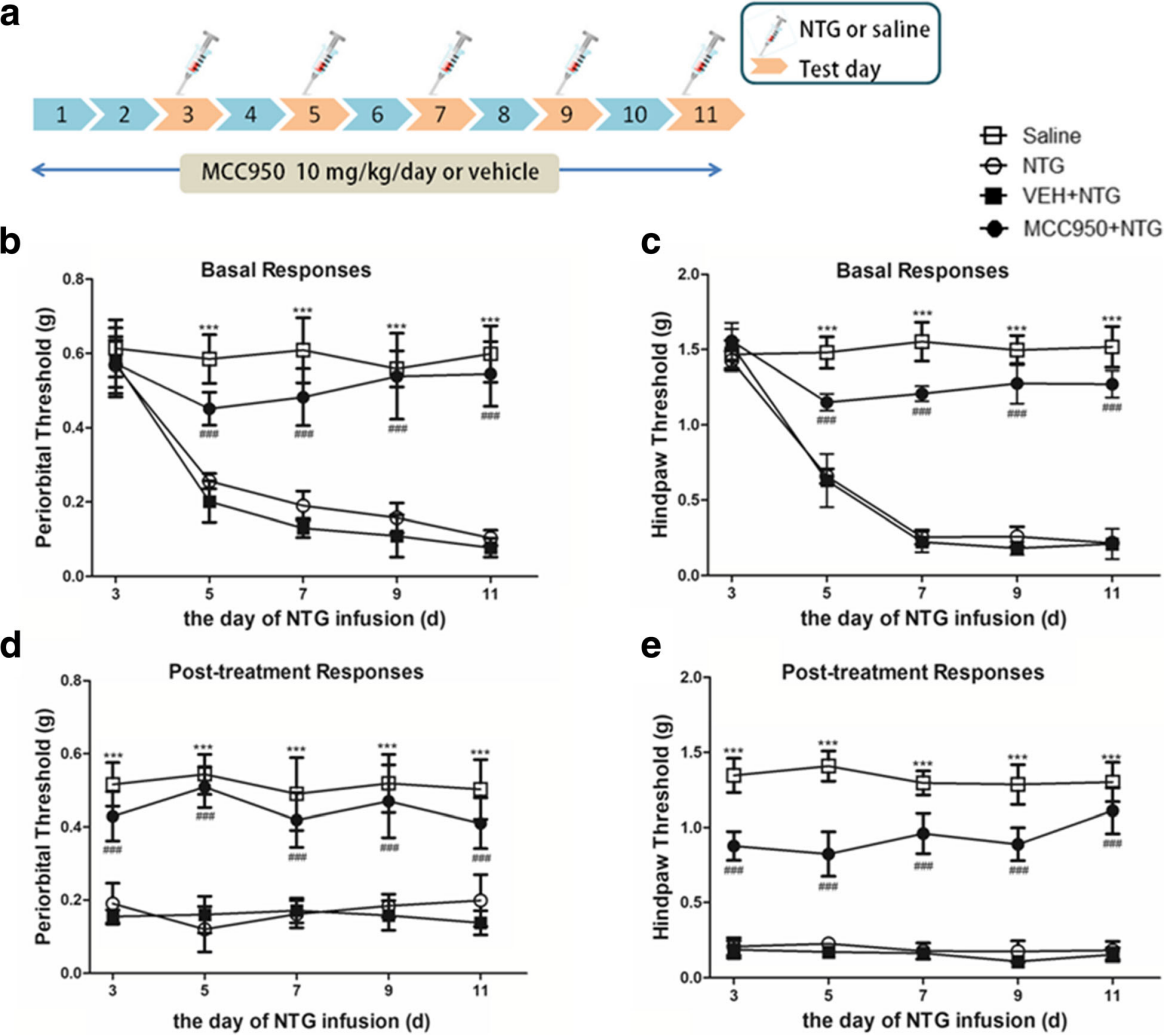
Chronic treatment with MCC950 (NLRP3 inhibitor) reversed NTG-induced hyperalgesia. **a** Drug administered in the second experiment. In treatment groups, mice were treated with VEH or MCC950 (10 mg/kg i.p.) daily for 11 days. On days 3, 5, 7, 9 and 11, basal threshold responses were determined, then mice were injected with VEH or MCC950; 30–45 min later, mice were injected with nitroglycerin (NTG; 10 mg/kg, i.p.). Acute treatment responses were assessed 2 h following NTG administration. The saline group and NTG group were injected with saline or NTG, respectively, and the mechanical sensitivity responses were also tested before injection and 2 h after injection. **b**, **c** Basal threshold responses were significantly decreased after NTG administrations. ***p < 0.001 for the saline group compared to the NTG group. In the groups receiving MCC950-preventative treatment, basal threshold responses induced by NTG was significantly reversed. ^###^*p* < 0.001 for the MCC950+NTG group compared to the NTG group. Two-way RM ANOVA and Holm-Sidak post hoc analysis (*n* = 8 mice per group). **d**, **e** NTG administrations induced acute hyperalgesia. ***p < 0.001 for the saline group compared to the NTG group. Acute NTG-induced hyperalgesia were blocked by treatment with MCC950. ^###^*p* < 0.001 for the MCC950+NTG group compared to the NTG group. Two-way RM ANOVA and Holm-Sidak post hoc analysis (*n* = 8 mice per group)

**Fig. 5:**
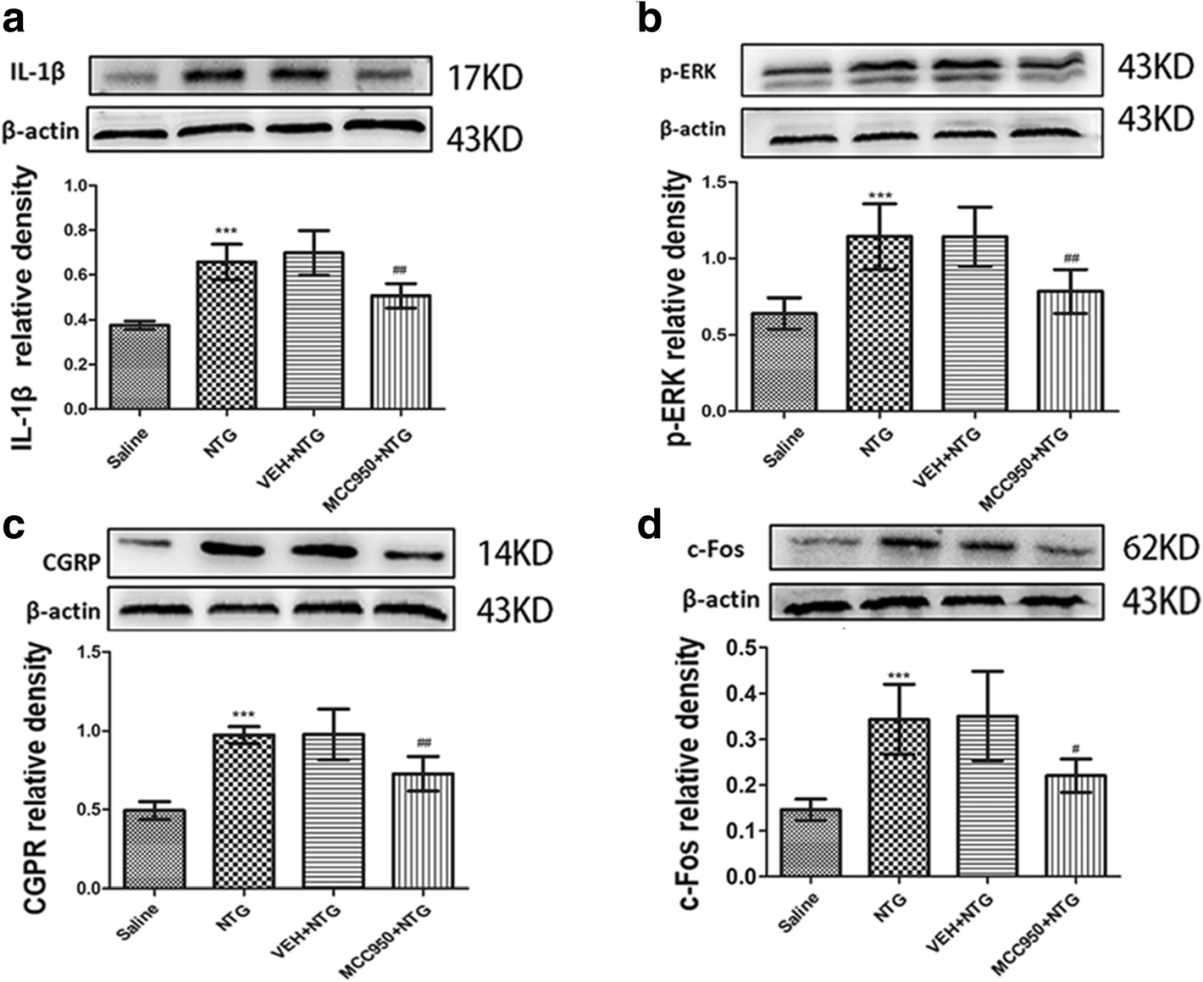
Chronic treatment with MCC950 (NLRP3 inhibitor) attenuated the increased expression levels of IL-1β, p-ERK, CGRP and c-Fos proteins. **a** NTG induced increase of IL-1β protein expression. Daily MCC950 preventative treatment decreased IL-1β protein expression. **b**–**d** The protein expression of p-ERK (**b**), CGRP (**c**) and c-Fos (**d**) upregulated in the NTG group compared to those in the saline group; compared with NTG, daily MCC950 preventative treatment decreased these protein levels. There were no obvious differences between the NTG and NTG+PBS groups. Statistical analyses were performed by one-way ANOVA, followed by a Tukey test; ***p < 0.001 vs. the saline group; ^##^*p* < 0.01, ^#^*p* < 0.05 vs. the NTG group (*n* = 6 mice per group)

In this experiment, mice received daily treatment with MCC950 (10 mg/kg, i.p.) or vehicle (equal volumes of PBS). On days 3, 5, 7, 9 and 11, mice were administered NTG (Fig. 4a). Daily treatment with MCC950 significantly inhibited NTG-induced basal hypersensitivity and acute hyperalgesia (*p* < 0.001; Fig. 4b–e). Compared with the NTG group, preventive treatment with NLRP3 inhibitor MCC950 not only significantly decreased the protein expression levels of IL-1β, but also decreased the protein expression levels of the CGRP, p-ERK and c-Fos in the TNC (*p* < 0.05; Fig. 5).

### IL-1β antagonist IL-1ra attenuated NTG-induced hyperalgesia and expression of CGRP, c-Fos and p-ERK in the TNC

**Fig. 6:**
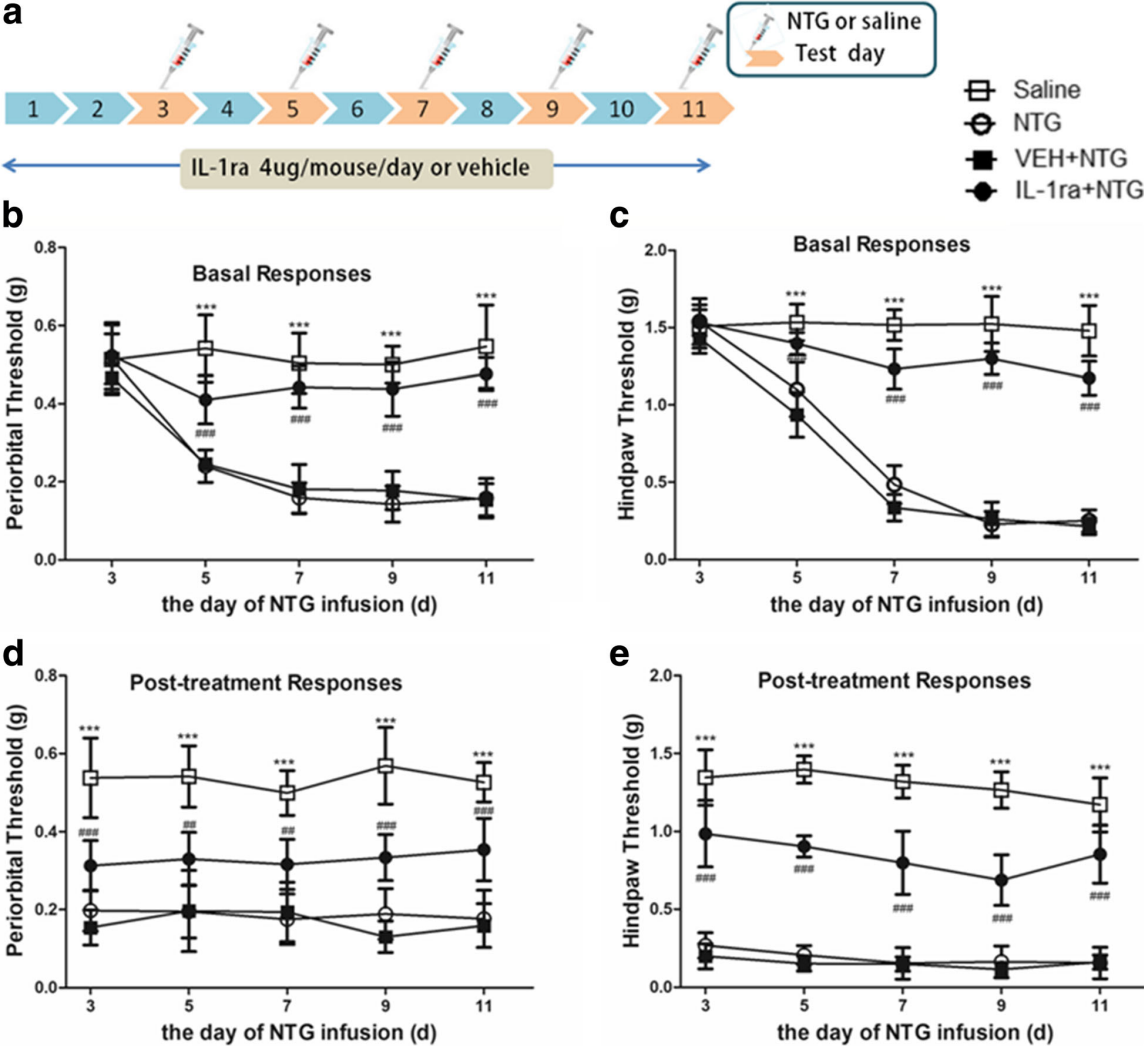
Chronic treatment with IL-1ra (IL-1β antagonist) reversed NTG-induced hyperalgesia. **a** Drug administered in the third experiment. In treatment groups, mice were treated with VEH or IL-1ra (4 μg/mouse, I.C.V.) daily for 11 days. On days 3, 5, 7, 9 and 11, basal threshold responses were determined, and then mice were injected with VEH or IL-1ra. 30–45 min later, mice were injected with nitroglycerin (NTG; 10 mg/kg, i.p.). Acute treatment responses were assessed 2 h following NTG administration. The saline group and NTG group just were injected saline or NTG, respectively, and the mechanical sensitivity responses were also tested before injection and 2 h after injection. **b**, **c** Basal threshold responses were significantly decreased after NTG administrations. ***p < 0.001 for the saline group compared to the NTG group. In the group receiving IL-1ra-preventative treatment, basal threshold responses induced by NTG significantly reversed. ^###^*p* < 0.001 for the IL-1ra+NTG group compared to the NTG group. Two-way RM ANOVA and Holm-Sidak post hoc analysis (*n* = 8 mice per group). **d**, **e** NTG injection induced acute hyperalgesia. ***p < 0.001 for the saline group compared to the NTG group. Acute NTG-induced hyperalgesia were blocked by treatment with IL-1ra. ^###^*p* < 0.001 for the IL-1ra+NTG group compared to the NTG group. Two-way RM ANOVA and Holm-Sidak post hoc analysis (*n* = 8 mice per group). I.C.V., intracerebroventricular

**Fig. 7:**
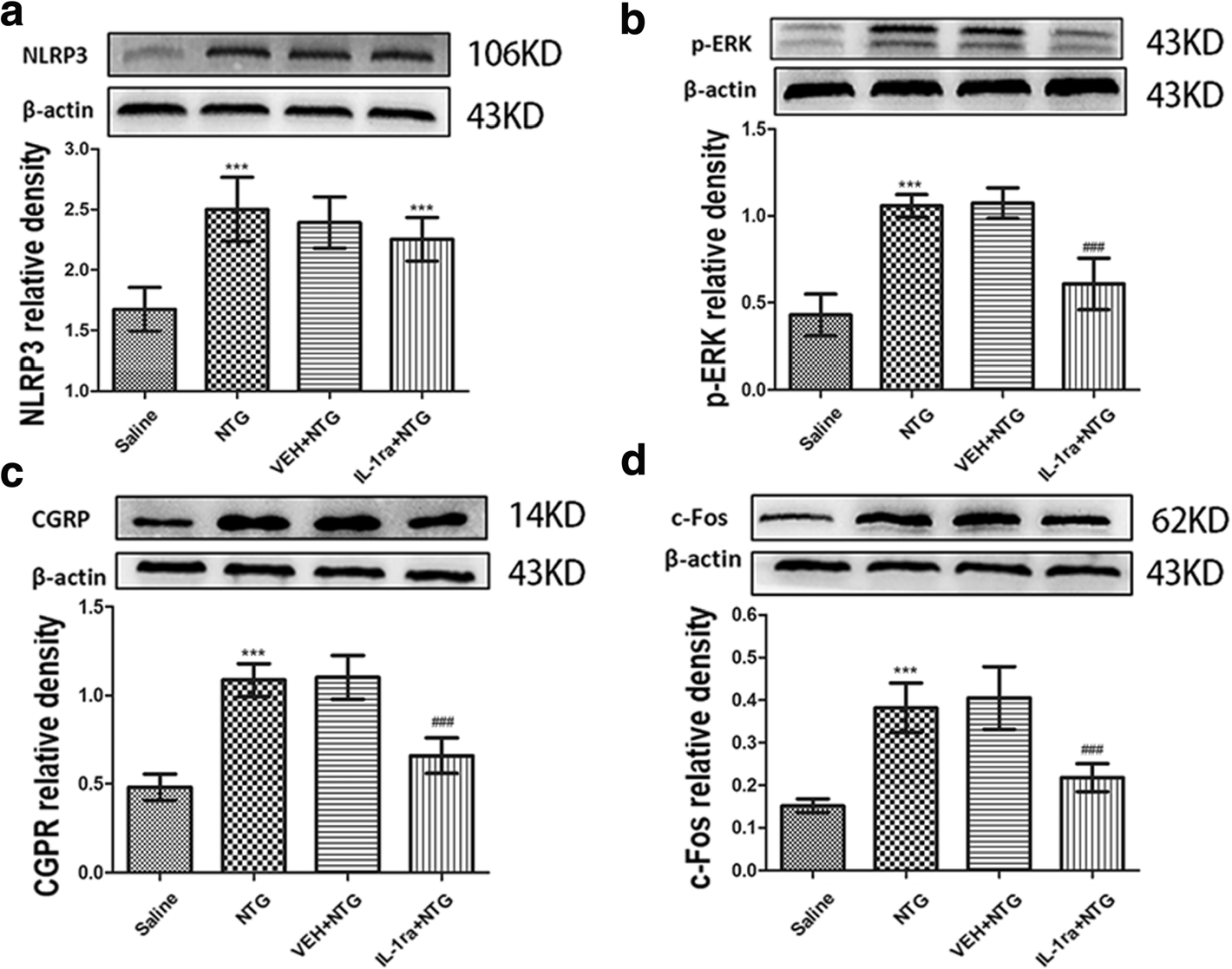
Chronic treatment with IL-1ra (IL-1β antagonist) attenuated increased expression levels of c-Fos, p-ERK and CGRP, but not effect on NLRP3. **a** Nitroglycerin (NTG) induced increase of NLRP3 protein expression. But NLRP3 expression had no significant differences between the daily IL-1ra preventative treatment group and NTG group. **b**–**d** The expression of p-ERK (**b**), CGRP (**c**) and c-Fos (**d**) showed upregulation in the NTG group compared to those in the saline group; compared with NTG, daily IL-1ra preventative treatment decreased these protein levels. There were no significant differences between the NTG and NTG+PBS groups. Statistical analyses were performed by one-way ANOVA, followed by a Tukey test; ***p < 0.001 vs. the saline group, ^###^*p* < 0.001 vs. the NTG group (*n* = 6 mice per group)

In this experiment, mice were received daily treatment with IL-1ra (4 μg/mouse/day, I.C.V.) or vehicle (equal volumes of PBS, I.C.V.) for 11 days. On days 3, 5, 7, 9 and 11, mice were administered NTG (Fig. 6a). Daily treatment with IL-1ra significantly inhibited NTG-induced basal hypersensitivity and acute hyperalgesia (Fig. 6b–e). Compared with the NTG group, preventive treatment with IL-1β antagonist IL-1ra significantly decreased the protein expression levels of CGRP; p-ERK and c-Fos were also significantly decreased (*p* < 0.001). However, the protein expression levels of NLRP3 were not significantly different from the NTG group (*p* > 0.05) (Fig. 7).

**Fig. 8:**
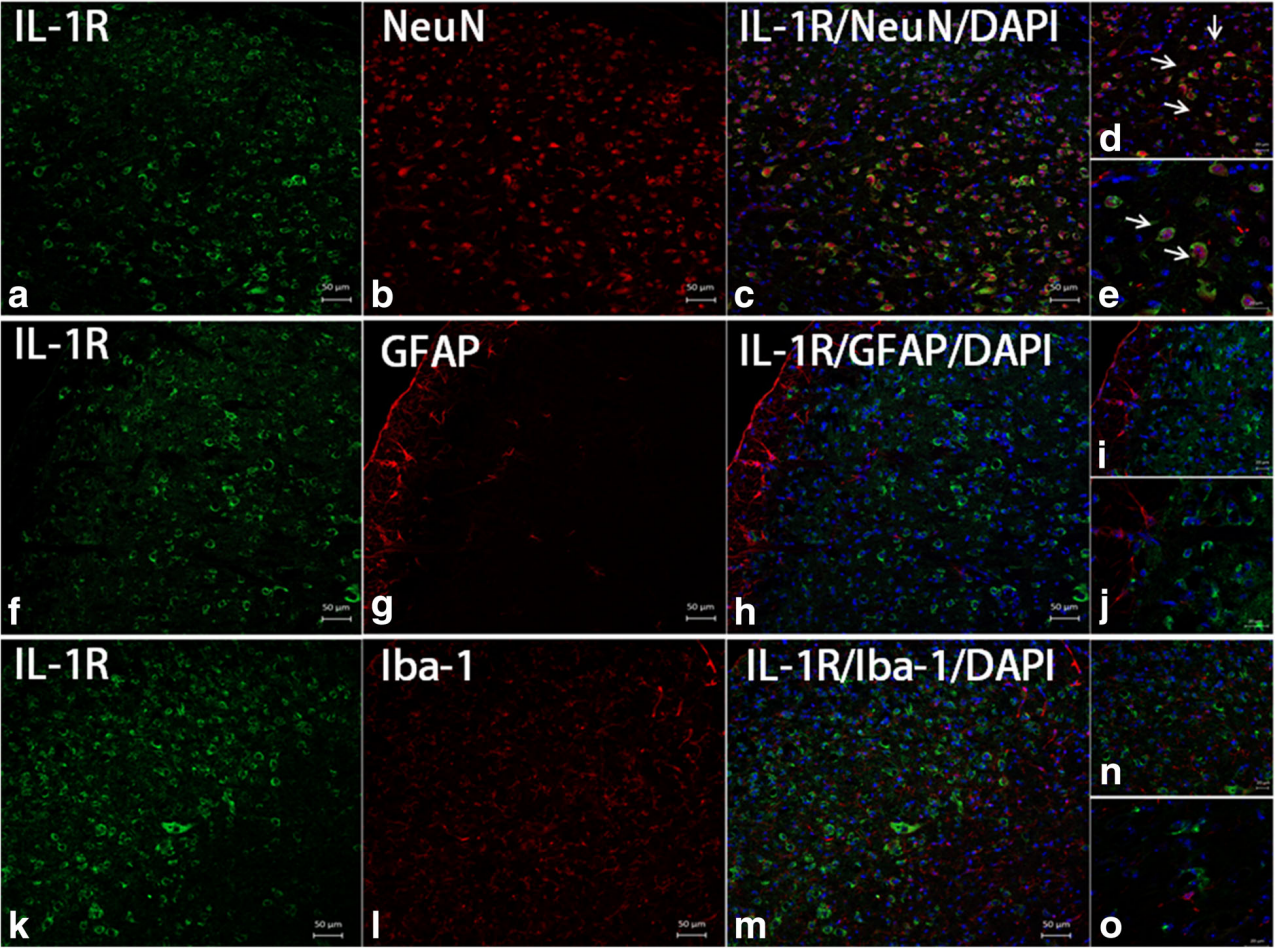
IL-1R was mainly expressed in the TNC neurons. Double immunofluorescence staining for IL-1R (green) with NeuN, a neuronal marker (red); GFAP, an astrocyte marker (red); or Iba-1, a microglial marker (red) in the mouse TNC after repeated nitroglycerin (NTG) administrations. **a**–**e** The results revealed the colocalization of IL-1R with NeuN in the TNC (white arrows). **f**–**o** The expression of IL-1R was not detected in GFAP- or Iba-1-positive cells. **a**–**c**, **f**–**h** and **k–m** × 10 objective lens; scale bars, 50 μm. **d**, **i** and **n** × 20 objective lens; scale bars, 20 μm. **e**, **j**, and **o** × 40 objective lens; scale bars, 20 μm

In addition, double immunostaining indicated that the IL-1β receptors, IL-1R was located mainly in neurons in the TNC (Fig. 8).

## Discussion

In this study, we used a published CM-associated animal model, which was established by repeated administration of the known human migraine trigger NTG in mice [25]. This model has been tested over the years with different migraine-related therapeutic drugs and is generally accepted as a reliable animal model of CM-associated pain [34].

Headache is a hallmark of migraine pain; hence, we tested hypersensitivity in the specific area that represents headache response, the periorbital area, which is innervated by the trigeminal nerve V1 branch. Repeated NTG stimulation produced acute hyperalgesia and a progressive basal hypersensitivity in the area. These results are consistent with those observed in NTG-induced migraine rat models [35]. Another study in mice found repeated stimulation of high affinity nitric oxide (NO) receptor, soluble guanylyl cyclase (sGC), also caused acute hyperalgesia and baseline hypersensitivity in the periorbital area [36]. NTG-induced migraine is related to its metabolite NO; therefore, the sGC stimulator VL-102 can replace NTG in the model [36]. In addition, Sol-Ji Kim reported the opposite result, which may be related to selecting different facial area; he tested whisker pads rather than periorbital area [37]. In addition to headache, CM patients may experience hyperalgesia of extracephalic areas, which is an outward manifestation of central sensitization [4, 38]. In this model, Repeated NTG stimulation also induced acute hyperalgesia and basal hypersensitivity in the hind paw, mimicking the characteristics observed in human CM patients. These results are consistent with those observed in rats or mice models that have been reported by other teams [25, 39].

The TNC, an important central area of the trigeminovascular system, regulates craniofacial pain. The increase in neuronal excitability in the TNC is related to the development of the central sensitization state [4]. In addition, NTG can activate neurons in the TNC area, and the increased excitability of these neurons was associated with NTG-induced hyperalgesia [35]. Therefore, we focused on the TNC as the region of interest to observe changes in biomarkers. c-Fos is a protein product of the immediate early gene and has been used extensively as a marker of neuronal activity. ERK is a member of the mitogen-activated protein kinase (MAPK) family, and phosphorylated ERK (p-ERK) is also always used as a marker of neuronal activation and central sensitization after tissue injury by noxious stimulation. Both c-Fos and p-ERK are rapidly and transiently induced following stimulation [32]. In our study, we examined the expression levels of c-Fos and p-ERK 2 h after the last NTG injection and found that the expression levels of both molecules were increased in the TNC. CGRP is considered an endogenous migraine generator and plays a critical role in the initiation and maintenance of migraine [40]. CGRP is synthesized and released in the superficial layer of the TNC by TG afferent fibres [41]. We detected whether the basal hypersensitivity induced by repeated NTG stimulation corresponded to an increase in CGRP expression in the TNC. Hence, we examined the CGRP expression level in the TNC 24 h after the last NTG injection and found that the repeated NTG stimulation increased the expression of CGRP in the TNC. The results of behavioural and biomarkers indicate that mice could develop a central sensitization of CM-associated pain after repeated NTG stimulation.

Our previous studies indicated that microglia activation contributes to central sensitization in CM-associated pain [9]. However, the specific mechanism involved in microglia is still unclear. This study found that NLRP3 was mainly expressed in microglia in the TNC. More importantly, we found that NTG-induced CM-associated pain is parallel with activation of NLRP3 inflammasomes for the first time. To further confirm the role of NLRP3 activation in migraine-associated pain, we tested the effects of the NLRP3 inhibitor MCC950 on the mice with repeated NTG stimulation. Daily MCC950 treatment not only significantly inhibited the acute hyperalgesia and basal hypersensitivity induced by NTG but also reduced the increased expression levels of p-ERK, c-Fos and CGRP induced by NTG.

The NLRP3 promotes the processing of pro-IL-1β to mature IL-1β [13]. Studies on the peripheral mechanism of neuropathic pain indicated that IL-1β induce peripheral sensitization of sensory neurons and mechanical hyperalgesia [42]. Recent research found that IL-1β could cause plasma extravasation in the rat cerebral cortex [43]. However, whether the IL-1β-induced plasma extravasation is related to vascular effects of migraine, there is no evidence right now. In the CNS, IL-1β plays a critical role in the regulation of microglia and neurons [14]. Our current results showed that the I.C.V. infusion with IL-1ra not only reverses mechanical hypersensitivity and the upregulated expression of CGRP, but also reverses upregulated expression of neuronal activation marker p-ERK and c-Fos. These data not only suggest that IL-1β contributes to central sensitization in CM-associated pain mice, but also its mechanism maybe related to microglial-neuronal pathway. Double IF staining revealed that in the TNC, IL-1β were mainly expressed in microglia, and its receptor, IL-1R, was mainly expressed in neurons. That provides some evidence for our hypothesis.

In recent years, the relationship between NLPR3 and pain has been gradually demonstrated. In the animal model of paclitaxel-induced pain, the NLRP3 inflammasome participates in the process of peripheral sensitization [42]. Based on another study, morphine tolerance is alleviated via inhibition of NLRP3 inflammasome activation in the microglia of the spinal dorsal horn [44]. However, few studies have investigated the relationship of NLRP3 with migraines. Only one study has shown the involvement of the NLRP3 inflammasome pathway in the peripheral TG response of the rat inflammatory dural stimulation-induced model of intracranial pain [45]. However, the role of the NLRP3 inflammasome pathway in the central area remains unknown. Our research fills this gap.

Nonetheless, which danger signal induces NLRP3 activation in subjects with CM has not been established. The combination of existing studies suggests that some mechanisms mentioned below may be involved in this process. The NLRP3 inflammasome is activated by the efflux of K+ through the open channel [46]. Purinergic receptor P2X, ligand-gated ion channel 7 receptor (P2X7R) is a nonselective cationic channel whose activation promotes the influx of Na+ and Ca2+ into the cytosol and the concomitant efflux of K+ [47]. Moreover, P2X7R is involved in the pathophysiological process of migraine, and antagonism of P2X7R by treatment with the specific P2X7 antagonist BBG leads to the alleviation of NTG-induced thermal hypersensitivity in mice [17]. Therefore, the activation of the NLRP3 receptor in CM may be related to P2X7R. NTG is bioactivated in the body to yield NO, which may react with peroxide in vivo and produce peroxynitrite (PN), which has been associated with migraine aetiology [19]. In recent years, researchers have found that the PN pathway plays an important role in the activation mode of NLRP3 [20]. Therefore, in the NTG-induced migraine-associated pain model, PN may be produced by NTG-derived NO; activate the NLRP3 molecular platform in microglia, leading to the release of downstream inflammatory mediators; and participate in the formation of central sensitization. Mitochondrial dysfunction and ROS production also reportedly trigger NLRP3 inflammasome activation [21]. Moreover, mitochondrial dysfunction and ROS production have been proven to be related to the cause of migraine [22, 48]. In the clinic, we usually perform preventative treatment of migraine using riboflavin (vitamin B2), which supports mitochondrial function, and vitamin C, which modulates the effects of ROS [49, 50].

The last topic worth mentioning is that drugs targeting the IL-1 downstream pathway of the inflammatory pathway have been developed. For example, anakinra (IL-1Ra) reportedly produces a good response to brain injury [51]. However, for some brain disorders with no damage or limited disruption of the blood-brain barrier, such as migraine, anakinra may not easily access the brain. The NLRP3 inflammasome is an available therapeutic target. To date, the most advanced NLRP3 small molecule inhibitor is MCC950 [29]. Our results confirm that MCC950 is protective in the NTG-induced migraine-associated pain model.

## Conclusions

**Fig. 9:**
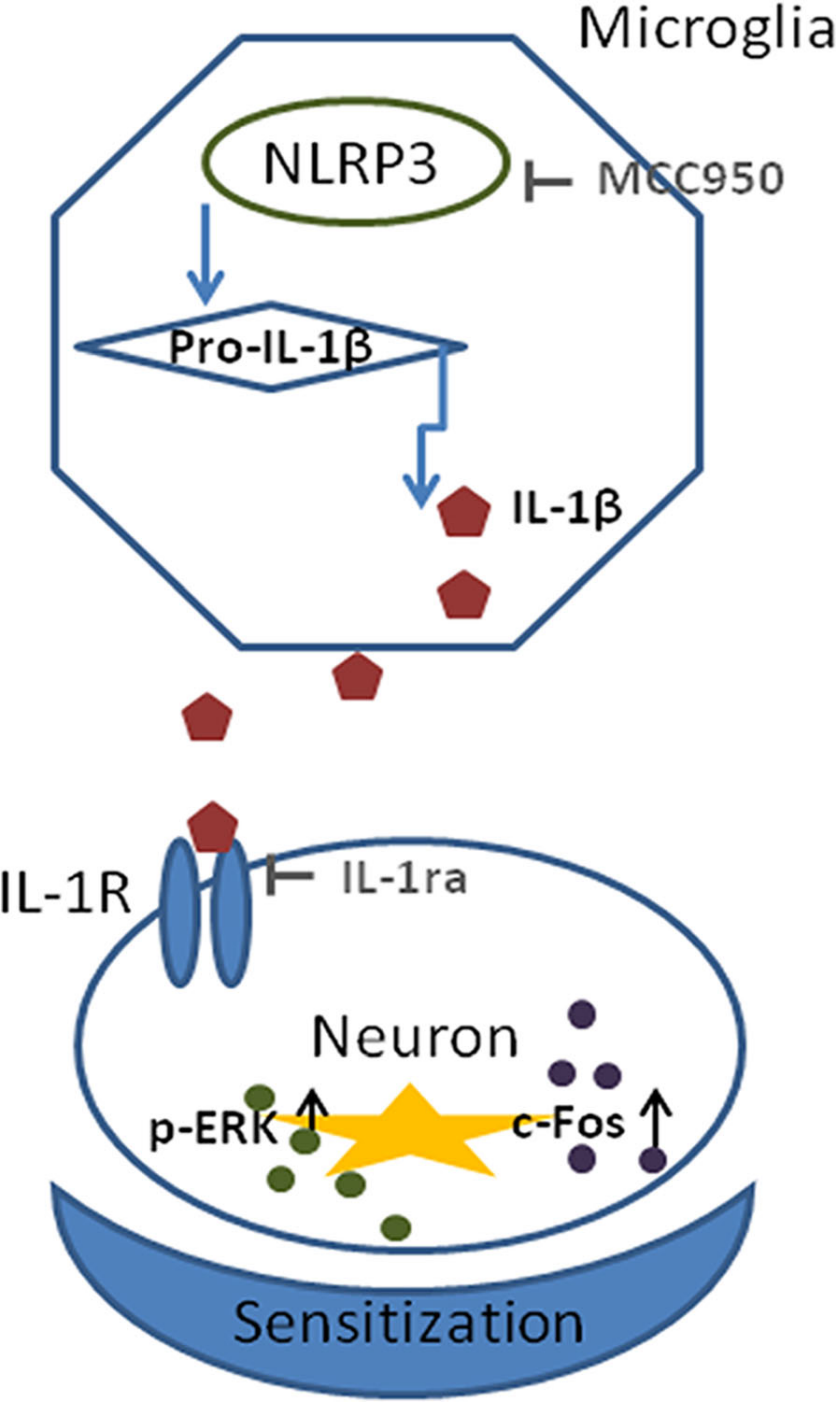
The NLRP3 pathway is a key regulator of the sensitization of a chronic migraine. Nitroglycerin (NTG) produces acute and chronic migraine-associated pain. In contrast, the NLRP3 inhibitor MCC950 blocks NTG-induced hyperalgesia. Blocking downstream IL-1β has a similar effect. These results indicate that NTG-associated pain is critically mediated by the NLRP3/IL-1β pathway. Our work indicates that inhibitors or negative modulators of the NLRP3/IL-1β pathway could be effective migraine treatments

Our study demonstrates that the expression of the NLRP3 inflammasome was upregulated in the migraine-associated pain mouse model that was induced by recurrent NTG stimulation. The increase in NLRP3 expression levels can activate IL-1β signalling. Blockade of NLRP3 and IL-1β both improved hyperalgesia and inhibited the increase in these biomarkers related to central sensitization of CM such as p-ERK, c-Fos and CGRP in the TNC (Fig. 9).

Together, these results provide new information regarding the physiopathology of CM. The NLRP3 inflammasome plausibly constitutes a target for the control of CM-associated pain, and its inhibition may represent a new therapeutic rationale and approach for migraine treatment.

## Additional files

Additional file 1: **Figure S1.** Scheme of the experimental design. (a) Experiment 1: Mice were randomly divided into the following groups: the nitroglycerin (NTG) group and saline group. (b) Experiment 2: Mice were randomly divided into the following groups: NTG, saline, NTG+VEH and NTG+MCC950 groups. Experiment 3: Mice were randomly divided into the following groups: NTG, saline, NTG+VEH, NTG+IL-1ra groups. VEH, vehicle; h, hour. (TIF 5765 kb)

Additional file 2: **Figure S2.** Repeated NTG administration increased the expression of microglial markers in the mouse TNC. (a) Coronal view showing the location of the TNC in the mouse brain. (b) Immunoreactive quantitative analysis of Iba-1 (microglial marker). Repeated nitroglycerin (NTG) or saline administered for 9 days. Compared to the saline group, the NTG group showed an increased ratio of the Iba-1-immunoreactive area to the total area of the TNC. Analyzed at × 10 images. Unpaired *t* test, ***p < 0.001 versus the saline group (*n* = 5 mice per group). (c, d) Immunostaining of the TNC for Iba-1 in the saline group and NTG group; nuclear staining was performed with DAPI (middle column). (c) × 10 objective lens; scale bar, 50 μm; (d) × 40 objective lens; scale bar, 20 μm. (TIF 3402 kb)

## Abbreviations

BCA: Bicinchoninic acid
BLAST: Basic local alignment search tool
CGRP: Calcitonin gene-related peptide
CM: Chronic migraine
CNS: Central nervous system
DAB: Diaminobenzidine
DAPI: 4,6-Diamidino-2-phenylindole
GAPDH: Glyceraldehyde 3-phosphate dehydrogenase
GFAP: Glial fibrillary acidic protein
I.C.V.: Intracerebroventricular
i.p.: Intraperitoneal
IF: Immunofluorescence
IgG: Immunoglobulin
IH: Immunohistochemistry
IL-1R: IL-1 receptor
IL-1ra: IL-1 receptor antagonist
IL-1β: Interleukin-1β
MAPK: Mitogen-activated protein kinase
*n*: Number in each experiment or group
NeuN: Neuronal nuclei
NLRP3: NOD-like receptor protein 3
NO: Nitric oxide
NTG: Nitroglycerin
OD: Optical density
P2X7: Purinergic receptor P2X, ligand-gated ion channel 7
PBS: Phosphate-buffered saline
p-ERK: Phosphorylated extracellular signal-regulated kinase
PFA: Paraformaldehyde
PN: Peroxynitrite
qRT-PCR: Quantitative real-time polymerase chain reaction
ROS: Reactive oxygen species
SD: Standard deviation
SDS PAGE: Sodium dodecyl sulfate polyacrylamide gel electrophoresis
sGC: Soluble guanylyl cyclase
TBS: Tris-buffered saline
TG: Trigeminal ganglion
TNC: Trigeminal nucleus caudalis
VEH: Vehicle
