# Supplementary figures and images for: Microglial NLRP3 inflammasome activation mediates IL-1β release and contributes to central sensitization in a recurrent nitroglycerin-induced migraine model
Source: J Neuroinflammation. 2019 Apr 10;16:78. doi: 10.1186/s12974-019-1459-7 (PMC6456991; doi:10.1186/s12974-019-1459-7)

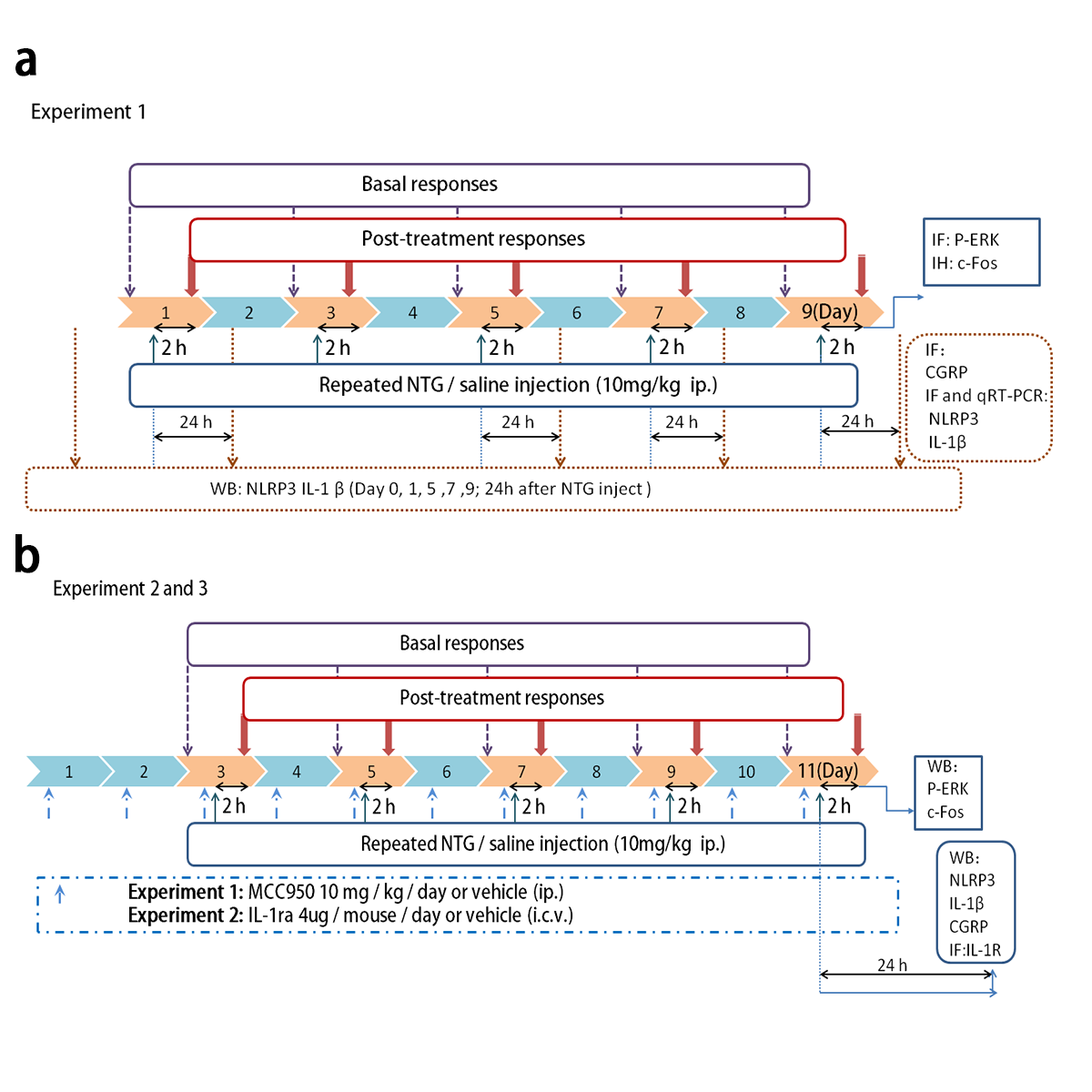

Supplement: Supplementary file 1 — Figure S1. Scheme of the experimental design. (a) Experiment 1: Mice were randomly divided into the following groups: the nitroglycerin (NTG) group and saline group. (b) Experiment 2: Mice were randomly divided into the following groups: NTG, saline, NTG+VEH and NTG+MCC950 groups. Experiment 3: Mice were randomly divided into the following groups: NTG, saline, NTG+VEH, NTG+IL-1ra groups. VEH, vehicle; h, hour. (TIF 5765 kb) [file 12974_2019_1459_MOESM1_ESM.tif]

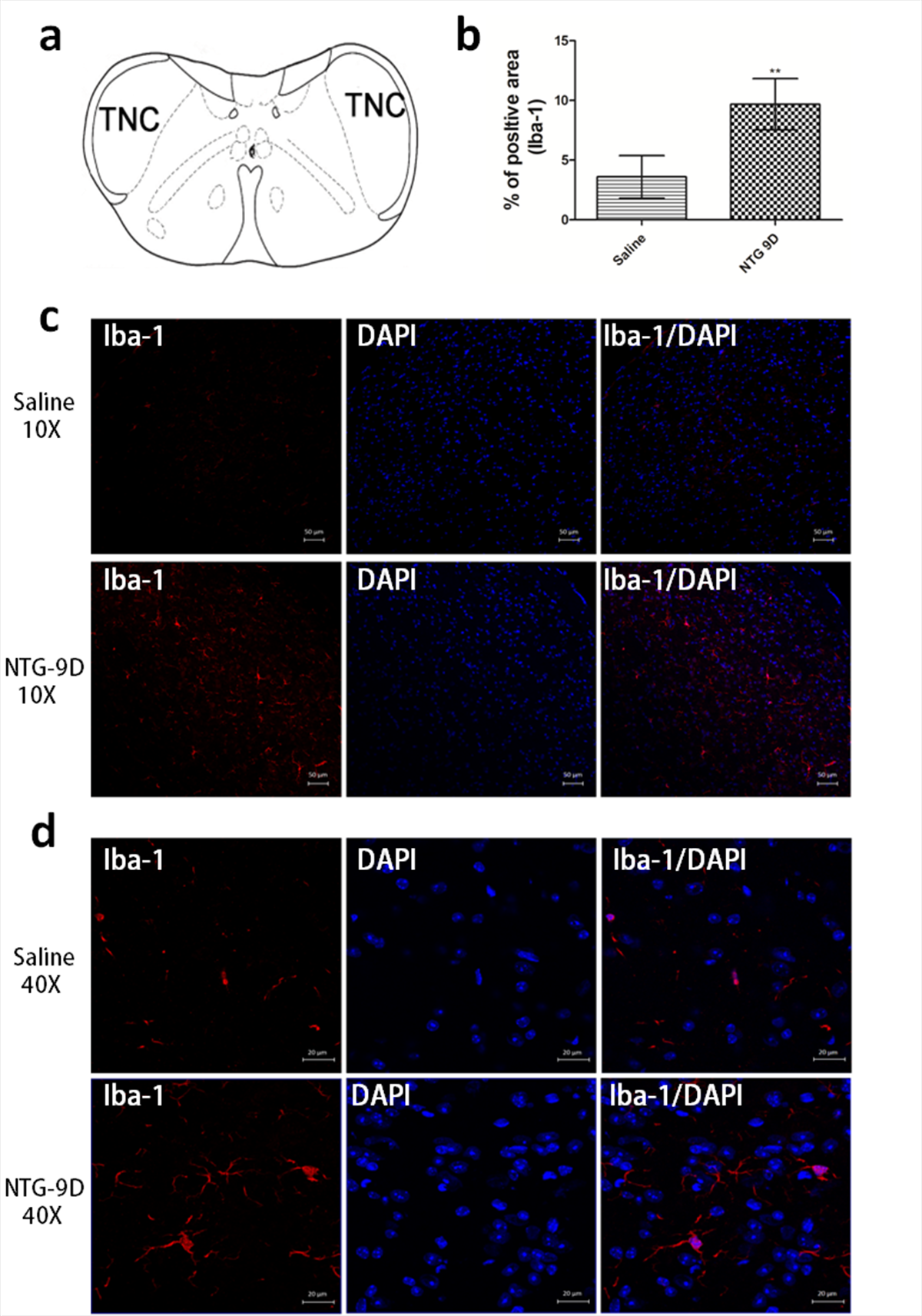

Supplement: Supplementary file 2 — Figure S2. Repeated NTG administration increased the expression of microglial markers in the mouse TNC. (a) Coronal view showing the location of the TNC in the mouse brain. (b) Immunoreactive quantitative analysis of Iba-1 (microglial marker). Repeated nitroglycerin (NTG) or saline administered for 9 days. Compared to the saline group, the NTG group showed an increased ratio of the Iba-1-immunoreactive area to the total area of the TNC. Analyzed at × 10 images. Unpaired t test, ***p < 0.001 versus the saline group (n = 5 mice per group). (c, d) Immunostaining of the TNC for Iba-1 in the saline group and NTG group; nuclear staining was performed with DAPI (middle column). (c) × 10 objective lens; scale bar, 50 μm; (d) × 40 objective lens; scale bar, 20 μm. (TIF 3402 kb) [file 12974_2019_1459_MOESM2_ESM.tif]
